# Supplementary material for: Characterization of a Cutibacterium acnes Camp Factor 1-Related Peptide as a New TLR-2 Modulator in In Vitro and Ex Vivo Models of Inflammation
Source: Int J Mol Sci. 2022 May 3;23(9):5065. doi: 10.3390/ijms23095065 (PMC9104286; doi:10.3390/ijms23095065)
Supplement: Supplementary file 1 [file ijms-23-05065-s001.zip › ijms-1707036-supplementary.pdf]

SUPPLEMENTARY MATERIAL

Tables and Figures

Tables

Table S1: CAMP1-related peptides sequences analyzed in peptide array

Peptide number	Location on glass slide	CAMP1 peptide sequence[a]
1	A 1	M-K-V-K-F-L-A-A-P-L-**V**-V-G-A-L
2	A 2	F-L-A-A-P-L-**V**-V-G-A-L-M-A-P-A
3	A 3	P-L-**V**-V-G-A-L-M-A-P-A-A-F-S-G
4	A 4	G-A-L-M-A-P-A-A-F-S-G-A-T-A-H
5	A 5	A-P-A-A-F-S-G-A-T-A-H-A-A-P-V
6	A 6	F-S-G-A-T-A-H-A-A-P-V-A-P-**M**-V
7	A 7	T-A-H-A-A-P-V-A-P-**M**-V-A-V-S-A
8	A 8	A-P-V-A-P-**M**-V-A-V-S-A-T-Q-P-N
9	A 9	P-**M**-V-A-V-S-A-T-Q-P-N-K-T-L-S
10	A10	V-S-A-T-Q-P-N-K-T-L-S-V-A-E-A
11	A11	Q-P-N-K-T-L-S-V-A-E-A-Q-K-E-L
12	A12	T-L-S-V-A-E-A-Q-K-E-L-Q-V-V-N
13	A13	A-E-A-Q-K-E-L-Q-V-V-N-A-R-I-A
14	A14	K-E-L-Q-V-V-N-A-R-I-A-S-L-L-D
15	A15	V-V-N-A-R-I-A-S-L-L-D-T-Q-K-S
16	A16	R-I-A-S-L-L-D-T-Q-K-S-A-K-E-A
17	A17	L-L-D-T-Q-K-S-A-K-E-A-F-A-P-A
18	A18	Q-K-S-A-K-E-A-F-A-P-A-N-V-**T**-N
19	A19	K-E-A-F-A-P-A-N-V-**T**-N-I-I-G-K
20	A20	A-P-A-N-V-**T**-N-I-I-G-K-L-L-E-T
21	A21	V-**T**-N-I-I-G-K-L-L-E-T-A-**K**-R-I
22	A22	I-G-K-L-L-E-T-A-**K**-R-I-K-E-A-L
23	A23	L-E-T-A-**K**-R-I-K-E-A-L-V-N-**I**-I
24	A24	**K**-R-I-K-E-A-L-V-N-**I**-I-K-G-G-**V**
25	B 1	E-A-L-V-N-**I**-I-K-G-G-**V**-A-F-L-K
26	B 2	N-**I**-I-K-G-G-**V**-A-F-L-K-S-I-P-T
27	B 3	G-G-**V**-A-F-L-K-S-I-P-T-R-V-E-L
28	B 4	F-L-K-S-I-P-T-R-V-E-L-L-V-T-M
29	B 5	I-P-T-R-V-E-L-L-V-T-M-V-D-T-V
30	B 6	V-E-L-L-V-T-M-V-D-T-V-N-G-A-A
31	B 7	V-T-M-V-D-T-V-N-G-A-A-H-T-L-Q
32	B 8	D-T-V-N-G-A-A-H-T-L-Q-D-K-A-Q
33	B 9	G-A-A-H-T-L-Q-D-K-A-Q-P-A-H-S
34	B10	T-L-Q-D-K-A-Q-P-A-H-S-H-V-F-L
35	B11	K-A-Q-P-A-H-S-H-V-F-L-E-L-V-H
36	B12	A-H-S-H-V-F-L-E-L-V-H-A-S-V-L
37	B13	V-F-L-E-L-V-H-A-S-V-L-L-V-**A**-V
38	B14	L-V-H-A-S-V-L-L-V-**A**-V-S-A-T-S
39	B15	S-V-L-L-V-**A**-V-S-A-T-S-D-Q-L-K
40	B16	V-**A**-V-S-A-T-S-D-Q-L-K-D-E-M-A

41	B17	A-T-S-D-Q-L-K-D-E-M-A-A-V-K-K
42	B18	Q-L-K-D-E-M-A-A-V-K-K-A-L-A-E
43	B19	E-M-A-A-V-K-K-A-L-A-E-A-Q-K-M
44	B20	V-K-K-A-L-A-E-A-Q-K-M-P-D-I-K
45	B21	L-A-E-A-Q-K-M-P-D-I-K-P-N-D-G
46	B22	Q-K-M-P-D-**I**-K-P-N-D-**G**-A-T-F-Y
47	B23	D-**I**-K-P-N-D-**G**-A-T-F-Y-T-K-**A**-K
48	B24	N-D-**G**-A-T-F-Y-T-K-**A**-K-L-**A**-R-V
49	C 1	T-F-Y-T-K-**A**-K-L-**A**-R-V-L-R-Q-I
50	C 2	K-**A**-K-L-**A**-R-V-L-R-Q-I-R-F-D-R
51	C 3	**A**-R-V-L-R-Q-I-R-F-D-R-N-T-C-V
52	C 4	R-Q-I-R-F-D-R-N-T-C-V-L-P-F-K
53	C 5	F-D-R-N-T-C-V-L-P-F-K-R-L-G-T
54	C 6	T-C-V-L-P-F-K-R-L-G-T-I-Y-F-**L**
55	C 7	P-F-K-R-L-G-T-I-Y-F-**L**-S-R-A-L
56	C 8	L-G-T-I-Y-F-**L**-S-R-A-L-L-K-A-T
57	C 9	Y-F-**L**-S-R-A-L-L-K-A-T-G-V-L-M
58	C10	R-A-L-L-K-A-T-G-V-L-M-E-P-L-V
59	C11	K-A-T-G-V-L-M-E-P-L-V-R-V-S-E
60	C12	V-L-M-E-P-L-V-R-V-S-E-V-D-Q-A
61	C13	P-L-V-R-V-S-E-V-D-Q-A-I-T-D-V
62	C14	V-S-E-V-D-Q-A-I-T-D-V-K-A-A-Y
63	C15	D-Q-A-I-T-D-V-K-A-A-Y-Q-D-A-L
64	C16	T-D-V-K-A-A-Y-Q-D-A-L-K-A-P-N
65	C17	A-A-Y-Q-D-A-L-K-A-P-N-R-L-L-T
66	C18	D-A-L-K-A-P-N-R-L-L-T-P-A-V-P
67	C19	A-P-N-R-L-L-T-P-A-V-P-**A**-V-C-A
68	C20	L-L-T-P-A-V-P-**A**-V-C-**A**-P-A-P-A
69	C21	T-P-**A**-V-P-A-V-C-**A**-P-A-P-A-A-S
70	C22	M-K-V-K-F-L-A-A-P-L-I-V-G-A-L
71	C23	F-L-A-A-P-L-I-V-G-A-L-M-A-P-A
72	C24	P-L-I-V-G-A-L-M-A-P-A-A-F-S-G
73	D 1	F-S-G-A-T-A-H-A-A-P-V-A-P-I-V
74	D 2	T-A-H-A-A-P-V-A-P-I-V-A-V-S-A
75	D 3	A-P-V-A-P-I-V-A-V-S-A-T-Q-P-N
76	D 4	P-I-V-A-V-S-A-T-Q-P-N-K-T-L-S
77	D 5	Q-K-S-A-K-E-A-F-A-P-A-N-V-L-N
78	D 6	K-E-A-F-A-P-A-N-V-L-N-I-I-G-K
79	D 7	A-P-A-N-V-L-N-I-I-G-K-L-L-E-T
80	D 8	V-L-N-I-I-G-K-L-L-E-T-A-R-R-I
81	D 9	I-G-K-L-L-E-T-A-R-R-I-K-E-A-L
82	D10	L-E-T-A-R-R-I-K-E-A-L-V-N-V-I
83	D11	R-R-I-K-E-A-L-V-N-V-I-K-G-G-I
84	D12	E-A-L-V-N-V-I-K-G-G-I-A-F-L-K
85	D13	N-V-I-K-G-G-I-A-F-L-K-S-I-P-T
86	D14	G-G-I-A-F-L-K-S-I-P-T-R-V-E-L
87	D15	V-F-L-E-L-V-H-A-S-V-L-L-V-T-V
88	D16	L-V-H-A-S-V-L-L-V-T-V-S-A-T-S
89	D17	S-V-L-L-V-T-V-S-A-T-S-D-Q-L-K
90	D18	V-T-V-S-A-T-S-D-Q-L-K-D-E-M-A

91	D19	V-K-K-A-L-A-E-A-Q-K-M-P-D-L-K
92	D20	L-A-E-A-Q-K-M-P-D-L-K-P-N-D-V
93	D21	Q-K-M-P-D-L-K-P-N-D-V-A-T-F-Y
94	D22	D-L-K-P-N-D-V-A-T-F-Y-T-K-T-K
95	D23	N-D-V-A-T-F-Y-T-K-T-K-L-S-R-V
96	D24	T-F-Y-T-K-T-K-L-S-R-V-L-R-Q-I
97	E 1	K-T-K-L-S-R-V-L-R-Q-I-R-F-D-R
98	E 2	S-R-V-L-R-Q-I-R-F-D-R-N-T-C-V
99	E 3	T-C-V-L-P-F-K-R-L-G-T-I-Y-F-M
100	E 4	P-F-K-R-L-G-T-I-Y-F-M-S-R-A-L
101	E 5	L-G-T-I-Y-F-M-S-R-A-L-L-K-A-T
102	E 6	Y-F-M-S-R-A-L-L-K-A-T-G-V-L-M
103	E 7	A-P-N-R-L-L-T-P-A-V-P-S-V-C-L
104	E 8	L-L-T-P-A-V-P-S-V-C-L-P-A-P-A
105	E 9	T-P-A-V-P-S-V-C-L-P-A-P-A-A-S
106	E10	F-L-K-S-I-P-T-R-V-E-L-L-**L**-T-M
107	E11	I-P-T-R-V-E-L-L-**L**-T-M-V-D-T-V
108	E12	V-E-L-L-**L**-T-M-V-D-T-V-N-G-A-A
109	E13	**L**-T-M-V-D-T-V-N-G-A-A-H-T-L-Q
110	E14	R-I-A-S-L-L-D-T-Q-K-S-A-K-**K**-A
111	E15	L-L-D-T-Q-K-S-A-K-**K**-A-F-A-P-A
112	E16	Q-K-S-A-K-**K**-A-F-A-P-A-N-V-L-N
113	E17	K-**K**-A-F-A-P-A-N-V-L-N-I-I-G-K
114	E18	R-R-I-K-E-A-L-V-N-V-I-K-G-G-**V**
115	E19	E-A-L-V-N-V-I-K-G-G-**V**-A-F-L-K
116	E20	N-V-I-K-G-G-**V**-A-F-L-K-S-I-P-T
142	F22	**Bio**-A-A-N-W-S-H-P-Q-F-E-K-A-A[b]
143	F23	G-K-P-I-P-N-P-L-L-G-L-D-S-T[c]
144	F24	N-D-Y-K-D-D-D-D-K-G-A-A-A[d]

[a]15-mer peptides were designed from the non-mutated (Accession number AAS92206.1) and mutated (Accession number KX581410) CAMP1 protein sequences. Mutated amino acids are shown in red.

[b]Biotinylated peptide

[c]V5 epitope tag

[d]FLAG tag

Table S2: KEGG pathways and corresponding genes in skin explants stimulated with nmrCAMP1 and after CAMP1-related B2 peptide pretreatment

KEGG pathway	KEGG identifier	nmrCAMP1 stimulation			B2-peptide treated + nmrCAMP1 stimulation			Gene name
		Gene count	Gene Ratio (%)	*P* value	Gene count	Gene Ratio (%)	*P* value	
Cytokine-cytokine receptor interaction	hsa04060	26	10.24	4.94E-11	34	7.67	6.42E-12	CSF3, **IL1RN**, CSF2, CXCL8, **CSF1**, CXCL1, CXCL3, TNF, CXCL2, **CXCL5**, CCL4, CCL2, **IL36RN**, **IL13RA2**, **IL11**, **IL33**, TGFB2, **TGFB3**, CD70, **IL1R1, TNFSF15, IL36G**, IL16, **PPBP**, **INHBA**, NGF, **BMP2**, IL6, **CXCL12**, IL23A, IL1B, TNFSF9, IL7R, CCL26, *CCL4L2, CCL3L3, CSF2RB, CCL5, IL15RA, CCL20*
IL-17 signaling pathway	hsa04657	16	6.3	7.36E-11	17	3.84	5.88E-09	CSF3, CXCL8, CSF2, *MMP1*, *MMP3*, TNFAIP3, CXCL1, CXCL3, PTGS2, CXCL2, TNF, **MMP9**, **CXCL5**, IL6, IL1B, CCL2, FOSB, *CCL20*, *NFKBIA*
TNF signaling pathway	hsa04668	18	7.09	8.511E-12	18	4.06	1.18E-08	CSF2, **CSF1**, *MMP3*, **VEGFC**, TNFAIP3, CXCL1, TRAF1, CXCL3, PTGS2, CXCL2, TNF, **MMP9, CXCL5**, IL6, IL1B, IRF1, **CREB3L1**, CCL2, NFKB1, *CCL20, SELE, NFKBAI, CCL5, BIRC3*
MAPK signaling pathway	hsa04010	14	5.51	2.13E-03	27	6.09	1.80E-07	**CSF1, RASGRF2**, FGF2, TNF, **DUSP16, CACNA1G, MECOM, STMN1, FLNC, CD14, DUSP5,** TGFB2, ANGPT2, **TGFB3, IL1R1, DUSP1, HGF, IGF2, VEGFC, NFATC1**, HSPA2, NGF, EREG, GADD45G, **KITLG**, IL1B, EPHA2, *NFKB1*, *GADD45A, HSPA1B, HSPA1A*
NF-kappa B signaling pathway	hsa04064	19	7.48	1.90E-13	15	3.39	1.09E-06	CXCL8, **IL1R1**, TNFAIP3, CXCL1, TRAF1, CXCL3, PTGS2, CXCL2, TNF, GADD45G, **CXCL12**, IL1B, CCL4, **BCL2**, **CD14**, *NFKB1*, *DDX58, GADD45A, CLL4L2, TICAM1, NFKBIA, CYLD, BIRC3*
Hematopoietic cell lineage	hsa04640	6	2.36	3.11E-02	14	3.16	3.53E-06	**IL11**, CSF3, **ITGAM**, CSF2, **CSF1**, **IL1R1**, **ITGA1**, TNF, IL6, **KITLG**, IL1B, **CD14**, **CD36**, IL7R

Viral protein interaction with cytokine and cytokine receptor	hsa04061	14	5.51	1.91E-08	13	2.93	2.15E-05	CXCL8, **CSF1**, CXCL1, **PPBP**, CXCL3, CXCL2, TNF, **CXCL5**, IL6, **CXCL12**, CCL4, CCL2, CCL26, *CCL3*, *CCL20, CCL3L3, CCL4L2, CCL5*
NOD-like receptor signaling pathway	hsa04621	22	8.66	7.50E-12	14	3.16	1.99E-03	CXCL8, TNFAIP3, CXCL1, CXCL3, CXCL2, TNF, **P2RX7**, IL6, OAS2, IL1B, **BCL2**, **CCL2**, GBP1, GBP3, *OAS1*, *OAS3*, *STAT1, RIPK2, TICAM1, NFKBAI*, *CCL5, CCL2, GBP4, BIRC3*
C-type lectin receptor signaling pathway	hsa04625	10	3.94	8.94E-05	8	1.81	2.69E-02	IL6, **CALML5**, **IL23A**, IL1B, IRF1, **NFATC1**, PTGS2, TNF, *NFKB1*, *NFKBAI, CYLD, STAT1, IL23A*
FoxO signaling pathway	hsa04068	9	3.54	2.24E-03	9	2.03	3.11E-02	IL6, TGFB2, BCL6, **IRS1**, **TGFB3**, IL7R, SOD2, SGK1, GADD45G, *GADD45A, PLK2*
Cellular senescence	hsa04218	8	3.15	2.09E-02	10	2.26	3.16E-02	**LIN54**, IL6, TGFB2, CXCL8, **CALML5**, **TGFB3**, RASSF5, **NFATC1**, NBN, GADD45G, *NFKB1*, *GADD45A*
Chemokine signaling pathway	hsa04062	15	5.91	7.15E-06	11	2.48	4.40E-02	**GRK3**, **CXCL12**, CXCL8, CCL4, CCL2, CXCL1, **PPBP**, CXCL3, CXCL2, **CXCL5**, CCL26, *NFKB1*, *CCL3*, *STAT1, CCL20, CCL3L3, CCL4L2, CCL5, CCL2*
PI3K-Akt signaling pathway	hsa04151	/	/	n.s.	24	5.42	1.46E-04	**CSF3, ANGPT2, CSF1, IRS1, HGF, ITGA1, TNC, IGF2, FN1, VEGFC, NGF, FGF2, THBS1, EREG, IL6, KITLG, ITGA10, CREB3L1, BCL2, ITGA7, ITGB7, SGK1, IL7R, EPHA2**
ECM-receptor interaction	hsa04512	/	/	n.s.	9	2.03	3.20E-03	**FRAS1, ITGA10, ITGA1, TNC, FN1, ITGA7, ITGB7, CD36, THBS1**
PPAR signaling pathway	hsa03320	/	/	n.s.	8	1.81	4.95E-03	**FADS2, FABP4, FABP5, MMP1, ME1, LPL, PPARG, CD36**
Glycerolipid metabolism	hsa00561	/	/	n.s.	7	1.58	7.06E-03	**DGAT2, ALDH1B1, AKR1B1, LPL, PLPP3, GLA, PNLIPRP3**
Ras signaling pathway	hsa04014	/	/	n.s.	14	3.16	1.37E-02	**ANGPT2, CSF1, CALML5, RASGRF2, HGF, IGF2, VEGFC, PLA2G3, NGF, RASAL3, FGF2, KITLG, RASSF5, EPHA2**
Rap1 signaling pathway	hsa04015	/	/	n.s.	13	2.93	1.52E-02	**ITGAM, ANGPT2, CSF1, CALML5, HGF, VEGFC, NGF, FGF2, THBS1, KITLG, ADORA2A, RASSF5, EPHA2**
TGF-beta signaling pathway	hsa04350	/	/	n.s.	8	1.81	1.63E-02	**TGFB2, BMP2, TGFB3, FST, INHBA, THBS1, TNF, SMAD7**
Focal adhesion	hsa04510	/	/	n.s.	12	2.71	2.62E-02	**ITGA10, CAV1, HGF, ITGA1, BCL2, TNC, FN1, VEGFC, ITGA7, ITGB7, FLNC, THBS1**
Toll-like receptor signaling pathway	hsa04620	13	5.12	2.67E-07	/	/	n.s.	*CXCL8, STAT1, CCL3L3, CCL4L2, TICAM1, TNF, NFKB1, NFKBIA, IL6, IL1B, CCL5, CCL4, CCL3*

Cytosolic DNA-sensing pathway	hsa04623	8	3.15	1.67E-04	/	/	n.s.	*NFKBIA, IL6, DDX58, CCL5, IL1B, CCL4L2, CCL4, NFKB1*
Apoptosis	hsa04210	11	4.33	1.67E-04	/	/	n.s.	*NFKBIA, GADD45A, PMAIP1, CSF2RB, TRAF1, NGF, TNF, CTSS, NFKB1, GADD45G, BIRC3*
RIG-I-like receptor signaling pathway	hsa04622	8	3.15	2.67E-04	/	/	n.s.	*IFIH1, NFKBIA, CYLD, CXCL8, DDX58, ISG15, TNF, NFKB1*
Necroptosis	hsa04217	10	3.94	2.67E-03	/	/	n.s.	*CYLD, STAT1, IL1B, TNFAIP3, EIF2AK2, PYGM, TICAM1, TNF, H2AC18, BIRC3*
JAK-STAT signaling pathway	hsa04630	8	3.15	2.67E-02	/	/	n.s.	*IL15RA, CSF3, IL6, CSF2, STAT1, IL23A, CSF2RB, IL7R*
Th17 cell differentiation	hsa04659	6	2.36	4.67E-02	/	/	n.s.	*NFKBIA, IL6, STAT1, IL23A, IL1B, NFKB1*
Antigen processing and presentation	hsa04612	5	1.97	4.67E-02	/	/	n.s.	*HSPA2, TNF, CTSS, HSPA1B, HSPA1A*

Gene name : differentially expressed genes present in both conditions

Gene name : differentially expressed genes present in both conditions and validated in qPCR

Gene name : differentially expressed genes present in B2-peptide treated + nmrCAMP1 condition

Gene name : differentially expressed genes present in B2-peptide treated + nmrCAMP1 condition and validated in qPCR

Gene name : differentially expressed genes present in nmrCAMP1 condition

Gene name : differentially expressed genes present in nmrCAMP1 condition and validated in qPCR

n.s. : none significant

Table S3: Primer used in this study

Name	Forward	Reverse
CAMP1F1	CACCATGAAGGTTAAGTTCTTAGCAGCG	GGAGGCGGCCGGAGCAGG
IL-1β	TCCTGCGTGTTGAAAGATGATAA	CAAATCGCTTTTCCATCTTCTTC
CXCL8/IL-8	TCTTGGCAGCCTTCCTGATT	TTTCTGTGTTGGCGCAGTGT
TNF-α	GAGCACTGAAAGCATGATCC	CGAGAAGATGATCTGACTGCC
GM-CSF (CSF2)	GGAGCATGTGAATGCCATCCAG	CTGGAGGTCAAACATTTCTGAGAT
GAPDH	GCCACATCGCTCAGACAC	GCCCAATACGACCAAATCC
IL-10	TCTCCGAGATGCCTTCAGCAGA	TCAGACAAGGCTTGGCAACCCA

FIGURES

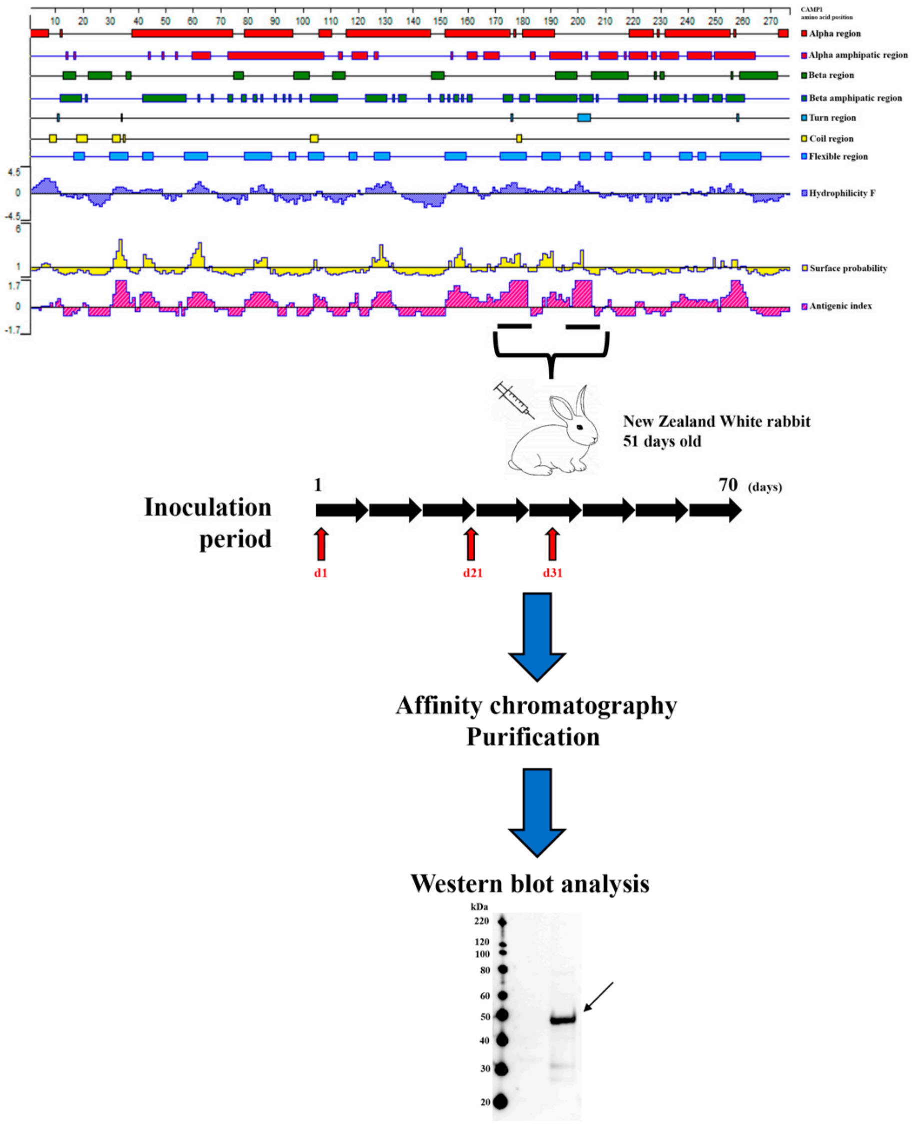

Figure S1: Polyclonal antibodies production against *C. acnes* CAMP factor 1. Two peptides from the *C. acnes* CAMP1 protein sequence (accession number AAS92206.1) with a high antigenic index (KMPDLKPNDVAT and VLRQIRFDRNTC) were injected three times over a period of 70 days in New Zealand white female rabbits. Antibodies against CAMP1 were purified by affinity chromatography. Western Blot analysis was performed on recombinant CAMP1 protein (50 µg) separated by electrophoresis in a 4–12% NuPAGE SDS BisTris gel and transferred onto nitrocellulose membrane and incubated with the polyclonal antibodies (10 µg/ml). The arrow indicates the detection of the recombinant CAMP1 (47 kDa).

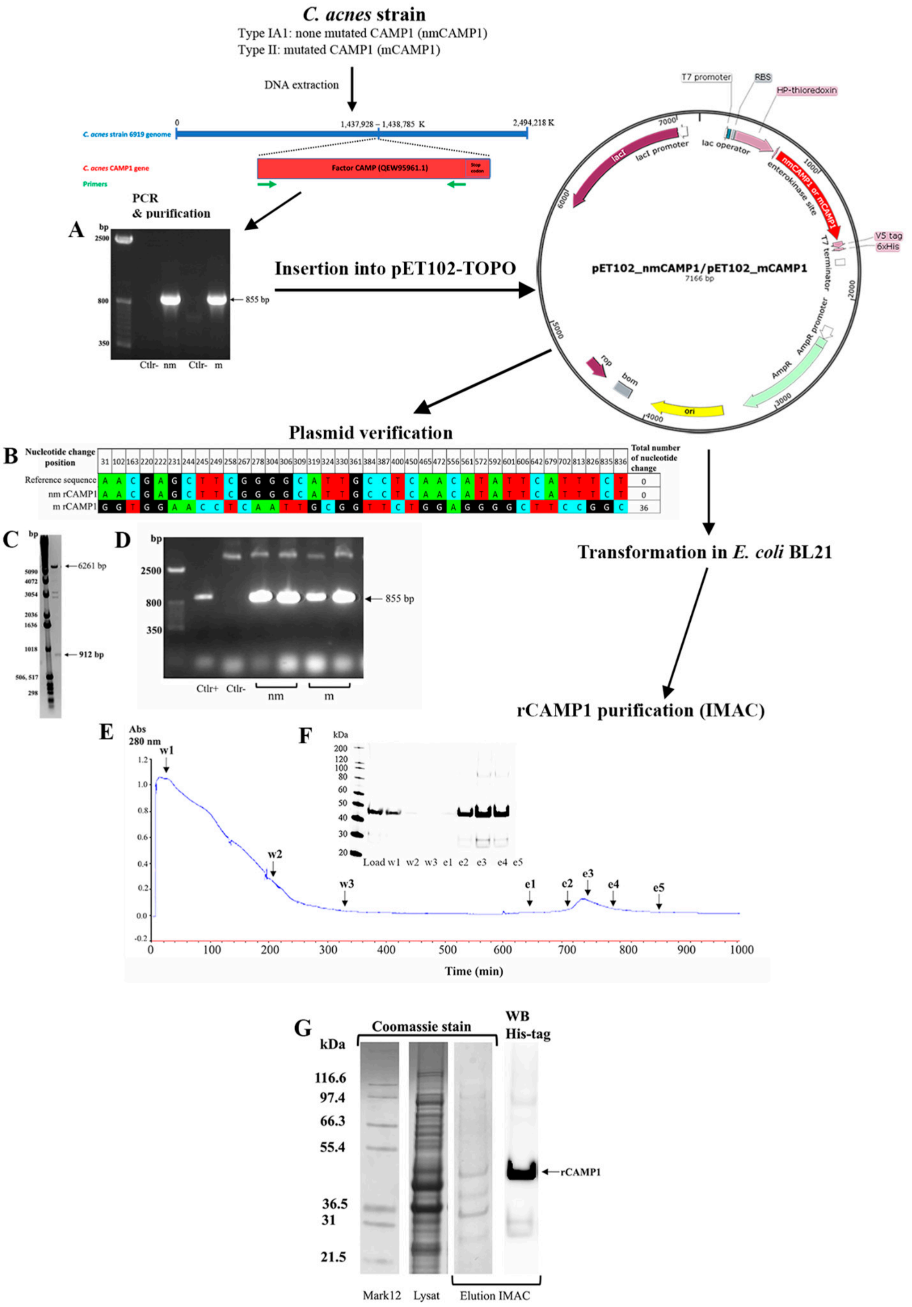

Figure S2: Cloning and production of *C. acnes* recombinant CAMP factor 1. (**A**) After DNA extraction from *C. acnes* strains of phylotypes IA1 (non-mutated CAMP1 or nmCAMP1) and II (mutated CAMP1 or mCAMP1), the CAMP1 genes were amplified by PCR with CAMP1F1 (forward and reverse) primers (Table S1). Amplicons (855 bp) were purified and inserted into the pET102D/TOPO expression vector to obtain the vectors pET102-nmCAMP1 and pET102-mCAMP1. The plasmid was verified by (**B**) sequencing; (**C**) enzymatic digestion with BamHI and ScaI, generating bands at 6261 and 912 bp, corresponding to the open plasmid and the fragment containing the CAMP1 amplicon, respectively; and (**D**) amplification. (**E-F**) Recombinant plasmids were introduced into the BL21 Star^{TM} (DE3) *E. coli* strain for overexpression. The bacterial lysate was loaded onto a Protino NI-TED resin IMAC column and the recombinant nmrCAMP1 and mrCAMP1 proteins containing a poly-histidine (6-His) tag at their N-terminal end were eluted.

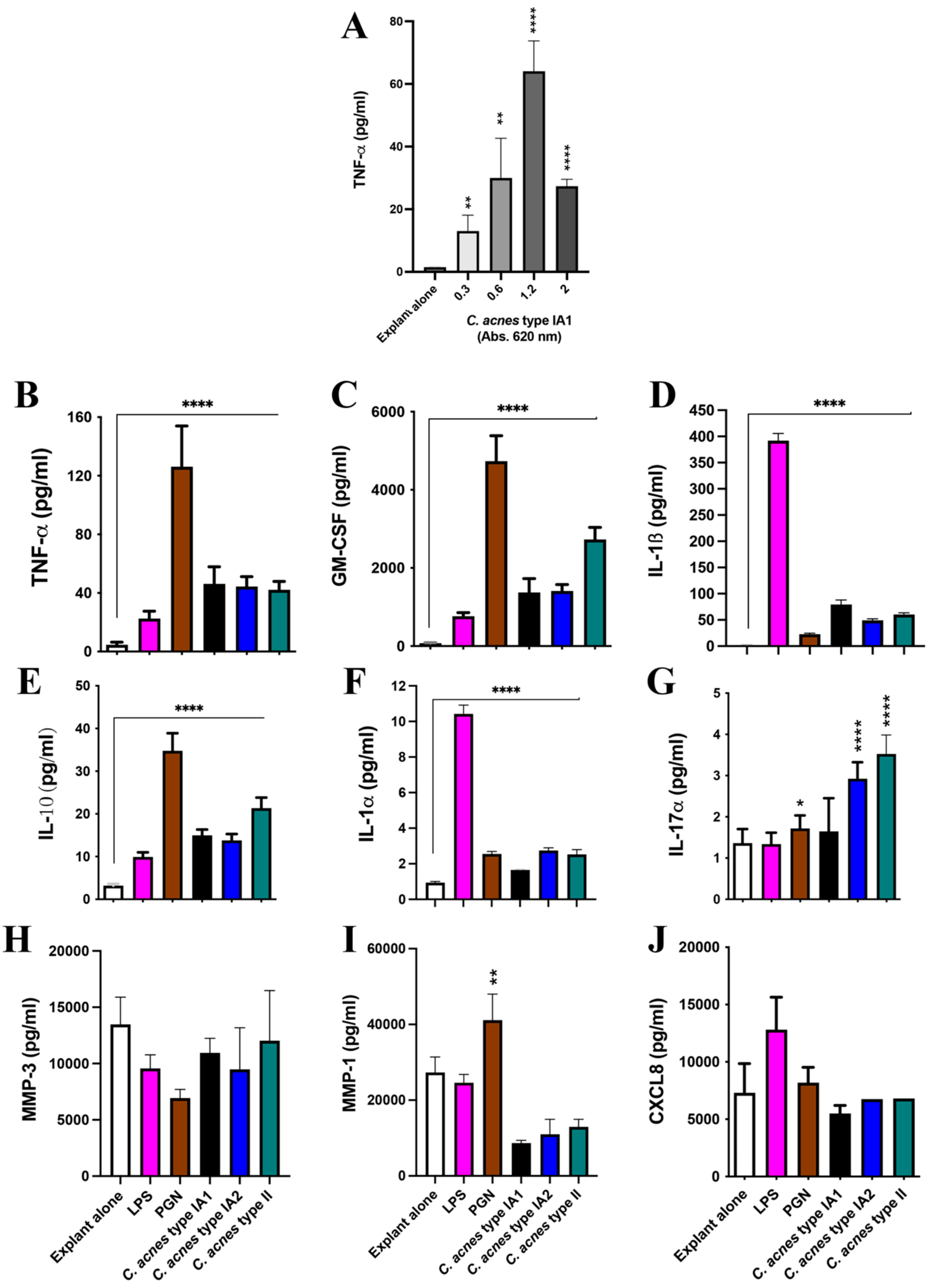

Figure S3: *C. acnes*-induced inflammation in *ex vivo* human skin explants. Human skin explants were left unstimulated (Explant alone) (white bar), (**A**) were stimulated with *C. acnes* suspensions at $OD_{620\ nm}$ values of 0.3 to 2.0 for 18 h (the data shown are means ± SEM, *n* = 3), or (**B**-**J**) were stimulated with LPS (5 µg/ml), PGN (5 µg/ml) and by *C. acnes* of various phylotypes (IA1, IA2 and II) at an $OD_{620\ nm}$ of 1.0 for 18 h (the data shown are means ± SEM, *n* = 6). (**A**) TNFα production was measured by ELISA in culture supernatant. (**B**-**J**) TNFα, GM-CSF, IL-1β, IL-10, IL-1α, IL-17α, MMP-3, MMP-1 and CXCL8/IL-8 levels were measured by Luminex in culture supernatant, respectively. Statistical significance is indicated by * (P<0.05), ** (P< 0.01), *** (P<0.001), and **** (P < 0.0001).

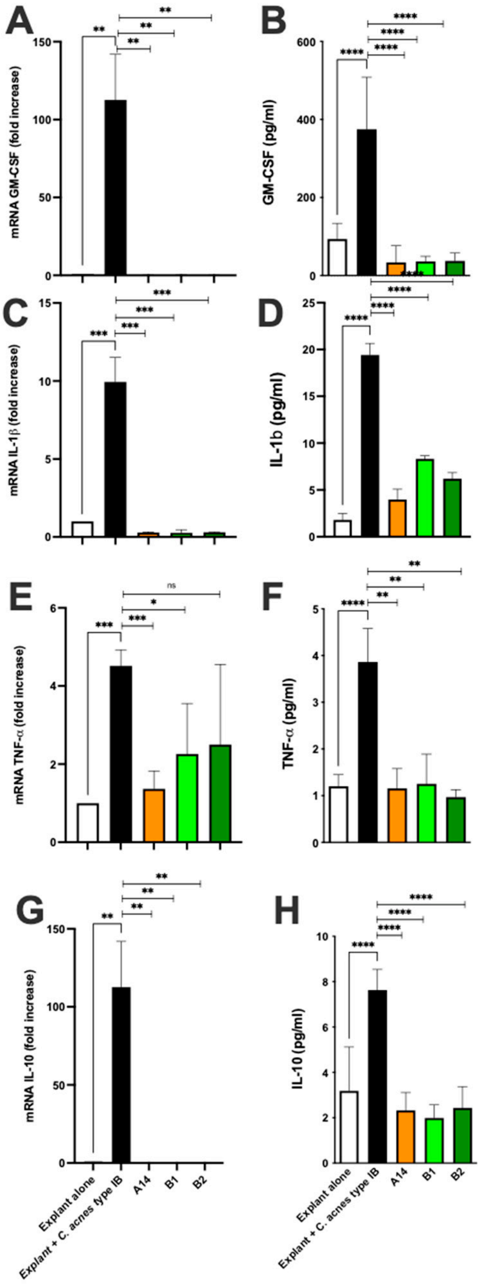

Figure S4: CAMP1-related peptides inhibit the production of *C. acnes*-induced proinflammatory molecules production *ex vivo*. Human skin explants were left untreated and unstimulated (Explant alone), stimulated by *C. acnes* type IB (O.D.$_{620\ nm}$ = 1.0) (Explant + *C. acnes* type IB), or were treated with A14, B1 and B2 peptide (62.5 µM) for 24 h and then stimulated by *C. acnes* for 18 h. (**A**, **C**, **E**, **G**) The levels of GM-CSF, IL-1β, TNF-α and IL-10 mRNA were measured by qRT-PCR. (**B**, **D**, **F**, **H**) The levels of GM-CSF, IL-1β, TNF-α and IL-10 productions were measured by Luminex in culture supernatant, respectively. The data shown are means ± SEM (n = 3). Statistical significance is indicated by * (P<0.05), ** (P< 0.01), *** (P<0.001), and **** (P < 0.0001).

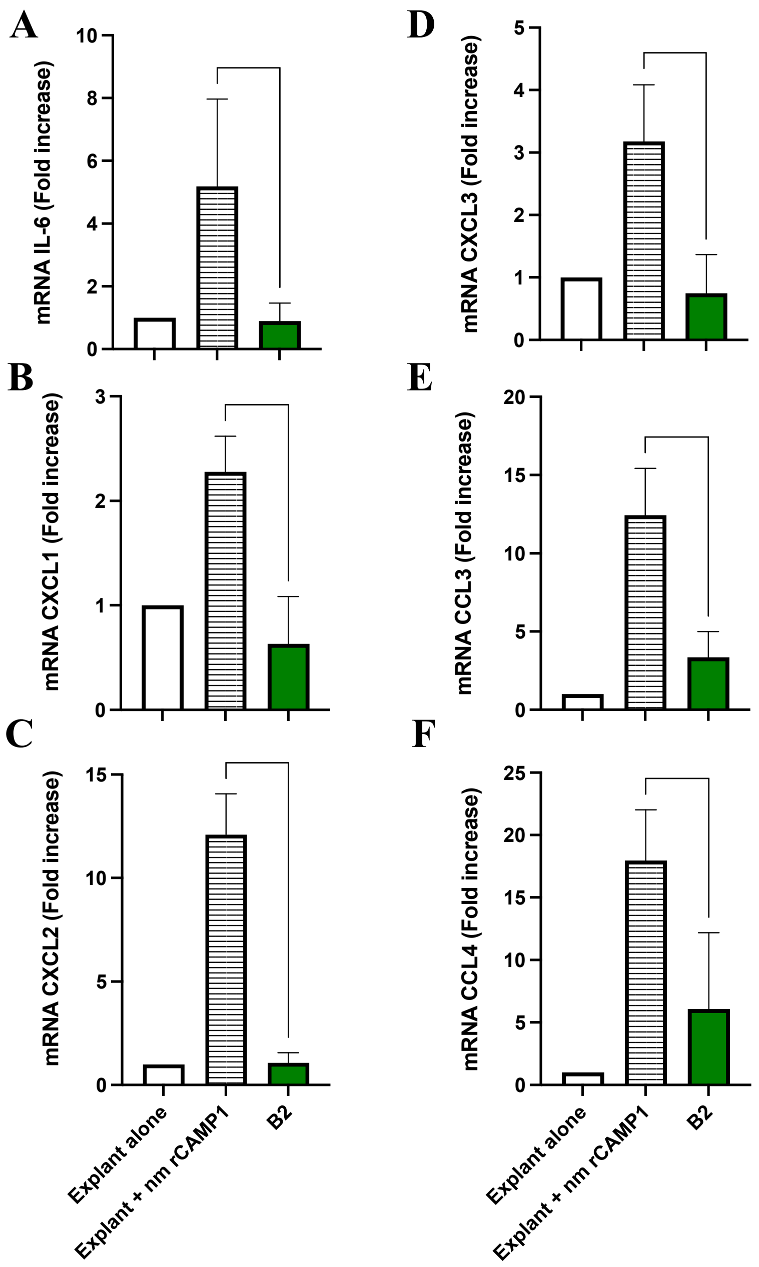

Figure S5: CAMP1-related B2 peptide inhibits the transcription of nmrCAMP1-induced proinflammatory molecules production *ex vivo*. Human skin explants were left untreated and unstimulated (Explant alone), were stimulated with nmrCAMP1 (Explant + nmrCAMP1), or were treated with B2 peptide (62.5 µM) for 24 h and then stimulated with nmrCAMP1 for 18 h. The levels of (**A**) IL6, (**B**) CXCL1, (**C**) CXCL2, (**D**) CXCL3, (**E**) CCL3, (**F**) CCL4 expression is assessed by qRT-PCR. The data shown are means ± SEM (n = 3). Statistical significance is indicated by * (P<0.05), ** (P< 0.01), *** (P<0.001), and **** (P < 0.0001).
